# LIMITED ENGLISH PROFICIENCY AND HEALTH LITERACY IN KOREAN OLDER ADULTS: MEDIATING EFFECT OF ACCULTURATION

**DOI:** 10.1093/geroni/igac059.1915

**Published:** 2022-12-20

**Authors:** Hae Sagong, Pao-Feng Tsai

**Affiliations:** Auburn University, Auburn, Alabama, United States; Auburn University, Auburn, Alabama, United States

## Abstract

Language proficiency and comprehension in the culture and health systems of the host country are imperative factors enabling appropriate health literacy (HL) for non-English speaking immigrants. Older immigrants with limited English proficiency have fewer opportunities and limited abilities to improve their English skills. Increasing individuals’ acculturation levels can be an effective strategy for older immigrants than solely educating English language skills. The purpose of this study is to investigate the mediating effect of acculturation between English proficiency and HL in older Korean immigrants. From June to October of 2020, a total of 244 older Korean immigrants aged 50 years or older residing in the Southern US were recruited. English proficiency was categorized into limited (very limited to fair) and fluent (fluent to very fluent). HL and acculturation were measured using the Health Literacy Survey-12 Questionnaires (HLS-Q12), and the East Asian Acculturation Measure (EAAM), respectively. Covariates were gender, age, monthly income, education, and length of residency in the U.S. The statistically significant differences were shown in age, gender, education, perceived health, and length of residency in the US by the level of English proficiency The limited English proficiency was negatively associated with HL (β= -.192, p=.002) and acculturation had a mediating effect between English proficiency and HL (β= -.133, p=.001). To alleviate the language barrier that causes low HL in older Korean immigrants, enhancing their understanding of the host country’s culture as well as improving organizational HL should be considered.

